# Supplementary material for: CD146 Delineates an Interfascicular Cell Sub-Population in Tendon That Is Recruited during Injury through Its Ligand Laminin-α4
Source: Int J Mol Sci. 2021 Sep 8;22(18):9729. doi: 10.3390/ijms22189729 (PMC8472220; doi:10.3390/ijms22189729)
Supplement: Supplementary file 1 [file ijms-22-09729-s001.zip › ijms-1357684-supplementary.pdf]

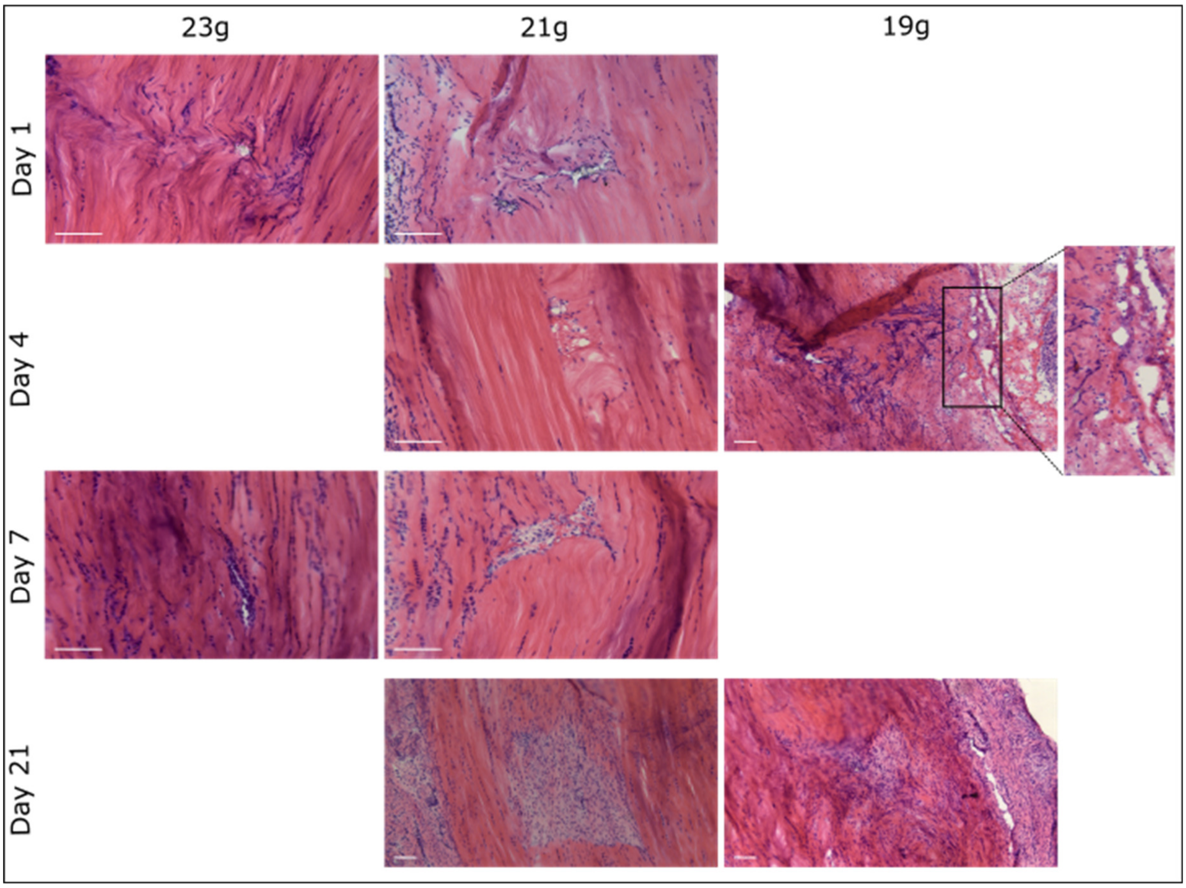

Figure S1. Optimisation of needle size and time points. Tendon puncture by 23G needles resulted in small lesions that were difficult to detect 7 days after injury. Use of 19G needles often resulted in damage to the edge of the tendon (see inset at day 4 post injury) and lesion size varied between individuals, particularly 21 days after injury. Using a 21G needle resulted in formation of a well-defined core lesion at all time points assessed, with less variation in lesion appearance between individuals. Scale bar = 100 µm. Note that injuries created by 23G and 19G needles were only assessed at days 1 & 7, and days 4 & 21 respectively to keep the number of animals used in preliminary experiments to a minimum.

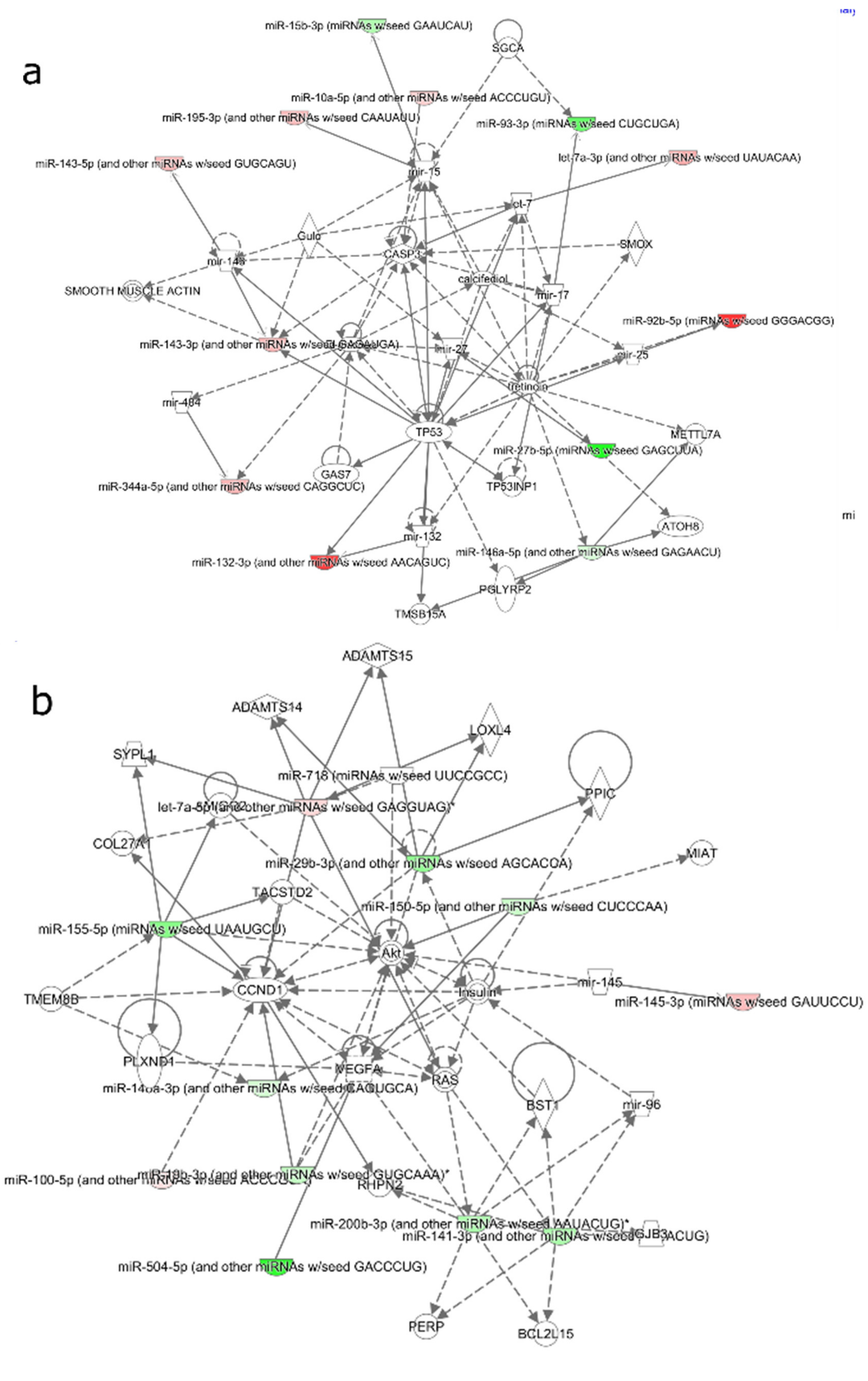
a
miR-15b-3p (miRNAs w/seed GAAUCAU)
SGCA
miR-10a-5p (and other miRNAs w/seed ACCCUGU)
miR-195-3p (and other miRNAs w/seed CAAUAUU)
miR-93-3p (miRNAs w/seed CUGCUGA)
let-7a-3p (and other miRNAs w/seed UAUACAA)
miR-143-5p (and other miRNAs w/seed GUGCAGU)
mir-15
let-7
Gulo
CASP3
SMOX
mir-148
SMOOTH MUSCLE ACTIN
calcifediol
mir-17
miR-92b-5p (miRNAs w/seed GGGACGG)
miR-143-3p (and other miRNAs w/seed GAGAUGA)
mir-27
mir-25
tretinoin
mir-484
TP53
METTL7A
miR-27b-5p (miRNAs w/seed GAGCUUA)
GAS7
miR-344a-5p (and other miRNAs w/seed CAGGCUC)
TP53INP1
ATOH8
mir-132
miR-146a-5p (and other miRNAs w/seed GAGAACU)
miR-132-3p (and other miRNAs w/seed AACAGUC)
PGLYRP2
TMSB15A
b
ADAMTS15
ADAMTS14
LOXL4
SYPL1
miR-718 (miRNAs w/seed UUCCGCC)
PPIC
COL27A1
miR-29b-3p (and other miRNAs w/seed AGCACCA)
MIAT
TACSTD2
miR-150-5p (and other miRNAs w/seed CUCCCAA)
miR-155-5p (miRNAs w/seed UAAUGCU)
Akt
CCND1
Insulin
mir-145
miR-145-3p (miRNAs w/seed GAUUCCU)
TMEM8B
PLXND1
VEGFA
RAS
BST1
mir-96
RHPN2
miR-200b-3p (and other miRNAs w/seed AAUACUG)*
GJB3
miR-504-5p (and other miRNAs w/seed GACCCUG)
PERP
BCL2L15

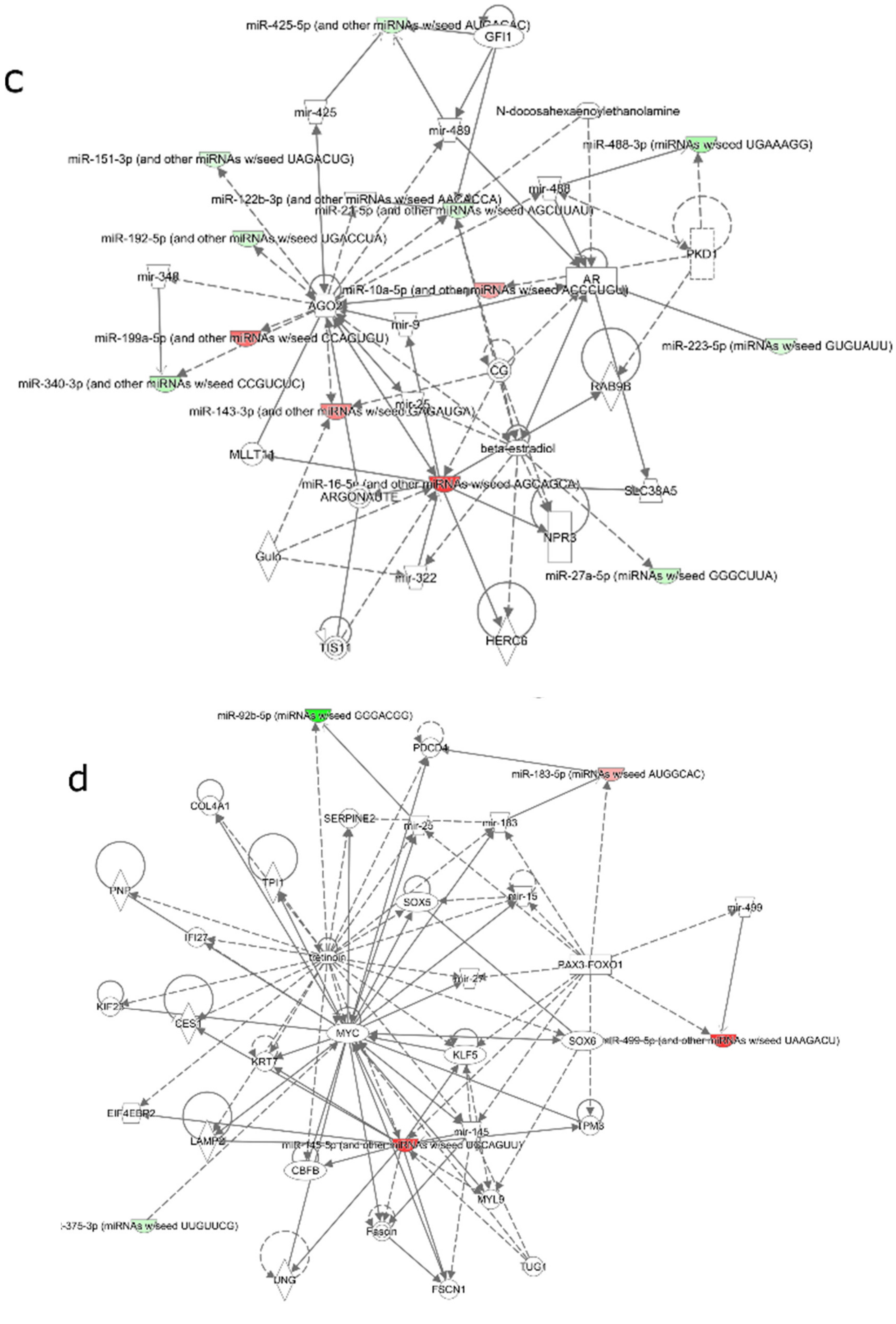

Figure S2. Top scoring networks derived from differentially expressed miRNAs in serum. Networks derived from differentially expressed miRNAs between sham and day 4 injured were associated with organismal injury and abnormalities (**a, b**), whereas those derived from differentially expressed miRNAs between day 4 and day 21 injured were associated with organismal injury and abnormalities (**c**) and cellular movement, cell death and survival (d). Red nodes, greater expression in sham (**a, b**) or day 4 injured (**c, d**); green nodes, greater expression in day 4 injured (**a, b**) or day 21 injured (**c, d**). Intensity of colour is related to higher fold-change.

Table S1. Modified Bonar scoring system used to grade injured, sham and contralateral control tendons.

	Grade			
	**0**	**1**	**2**	**3**
Cell morphology	Inconspicuous elongated spindle shaped nuclei with no obvious cytoplasm at light microscopy	Increased roundness: nucleus becomes more ovoid to round in shape without conspicuous cytoplasm	Increased roundness and size; the nucleus is round, slightly enlarged and a small amount of cytoplasm is visible	Nucleus is round, large with abundant cytoplasm and lacuna formation (chondroid change)
Cellularity	Mainly discrete cells	Hyper cellular, in rows and/or increased cell numbers	Areas of hypo as well as hyper cellularity	Area of assessment is mostly a-cellular
Vascularity	No vessels present in FOV/ Inconspicuous blood vessels coursing between bundles	Occasional cluster of vessel	2–3 clusters of capillaries/vessels	Areas with greater than 3 clusters
Tissue Organisation	Collagen fibres arranged linearly with cell nuclei aligned with long axis of tendon	Small loss of collagen and cell alignment in regions	Moderate loss of collagen and cell alignment	Complete loss of collagen and cell alignment, matrix appears disorganised

Table S2. Primary and secondary antibodies and blocking conditions used for immunolabelling.

**Primary Antibody**	**Supplier**	**Dilution**	**Secondary Antibody**	**Supplier**	**Dilution**	**Blocking conditions**
CD146	Abcam (ab75769)	1:100	Goat anti-rabbit IgG	Dako	1:500	5% goat & 5% rat serum; 1% BSA
Pan-Laminin	Abcam (ab11575)	1:100	Goat anti-rabbit IgG	Dako	1:500	5% goat & 5% rat serum; 1% BSA
Laminin-α4	St. John's Laboratories (STJ93891)	1:100	Goat anti-rabbit IgG	Dako	1:500	5% goat & 5% rat serum; 1% BSA
SDF-1	Abcam (ab9797)	1:100	Goat anti-rabbit IgG	Dako	1:500	5% goat & 5% rat serum; 1% BSA

CXCR-4	St. John's Laboratories (STJ97564)	1:100	Goat anti-rabbit IgG	Dako	1:500	5% goat & 5% rat serum; 1% BSA
CD14	Bioss (BS-1192R)	1:200	Goat anti-rabbit IgG	Dako	1:500	5% goat & 5% rat serum; 1% BSA
CD163	Bioss (BS-2527R-BSS)	1:400	Goat anti-rabbit IgG	Dako	1:500	5% goat & 5% rat serum; 1% BSA
CD86	R&D systems (AF1340)	1:45	Rabbit anti-goat IgG	Dako	1:500	1% BSA
CD31	Abcam (ab28364)	1:100	Goat anti-rabbit IgG	Dako	1:500	5% goat & 5% rat serum; 1% BSA
PCNA	St. John's Laboratories (STJ94982)	1:100	Goat anti-rabbit IgG	Dako	1:500	5% goat & 5% rat serum; 1% BSA
Collagen-III	St. John's Laboratories (STJ92388)	1:100	Goat anti-rabbit IgG	Dako	1:500	5% goat & 5% rat serum; 1% BSA
CD271	St. John's Laboratories (STJ94481)	1:100	Goat anti-rabbit IgG	Dako	1:500	5% goat & 5% rat serum; 1% BSA
TGF-β	St. John's Laboratories (STJ95997)	1:500	Goat anti-rabbit IgG	Dako	1:500	5% goat & 5% rat serum; 1% BSA
Scleraxis	Biorbyt (orb158355)	1:100	Goat anti-rabbit IgG	Dako	1:500	5% goat & 5% rat serum; 1% BSA

Table S3. Details of miRNA assays used to validate RNA-sequencing results.

**ID**	**Gene ID**	**miRNA sequence**	**Product Code**
let-7c-5p	MIMAT0000064	UGAGGUAGUAGGUUGUAUGGUU	YP00204767
miR-140-3p	MIMAT0004597	UACCACAGGGUAGAACCACGG	YP00204304
miR-103a-3p	MIMAT0000101	AGCAGCAUUGUACAGGGCUAUGA	YP00204063
miR-365-5p	MIMAT0017184	GAGGGACUUUCAGGGGC	YP02118503
miR-338-5p	MIMAT0004646	AACAAUAUCCUGGUGCUGAGUG	YP00204114
miR-143-3p	MIMAT0000849	UGAGAUGAAGCACUGUAGCUCA	YP00205106
miR-195-3p	MIMAT0017149	CCAAUAUUGGCUGUGCUGCUCCA	YP02103742
miR-672-5p	MIMAT0005327	UGAGGUUGGUGUACUGUGUGUGA	YP00205170
miR-218a-5p	MIMAT0000888_1	UUGUGCUUGAUCUAACCAUGU	YP00206034
